# Synthesis of nano-optical elements for zero-order diffraction 3D imaging

**DOI:** 10.1038/s41598-022-12414-y

**Published:** 2022-05-23

**Authors:** Alexander Goncharsky, Anton Goncharsky, Svyatoslav Durlevich, Dmitry Melnik

**Affiliations:** 1 Research Computer Center, M.V. Lomonosov Moscow State University, Leninskiye Gory, 1, Building 4, Moscow, Russia 119991; 2 Computer Holography Centre Ltd., Str. 2, Proezd 4922, Zelenograd, Moscow, Russia 124460; 3 Moscow Center for Fundamental and Applied Mathematics, 119991 Moscow, Russia

**Keywords:** Displays, Optics and photonics, Applied optics

## Abstract

A method is proposed to compute and synthesize a microrelief to produce a new nano-optical element for forming 3D images with full parallax at the zero order of diffraction. The synthesis of nano-optical elements requires the use of multilevel structures. A method is developed for the first time to compute the phase function of such nano-optical elements. Optical security elements that produce the new security feature are synthesized using electron-beam technology. The accuracy of microrelief formation is 10 nm in terms of depth. A sample optical security element is manufactured, which when illuminated by white light, forms a 3D image at the zero order of diffraction. Photos and video of the new 3D visual effect exhibited by real optical elements are presented. The optical elements developed can be replicated using standard equipment employed for manufacturing security holograms. The new optical security feature is easy to control visually, safely protected against counterfeiting, and designed to protect banknotes, documents, ID cards, etc.

## Introduction

The extensive development of methods for synthesizing optical elements to form 2D and 3D images began in the early 1970s after Denis Gabor was awarded the Nobel Prize for the invention and development of holographic recording techniques^1^. Gabor's followers^2^ developed methods for recording 3D holograms, which formed 3D images, when illuminated by a point light source. The technique developed became widely used in anti-counterfeiting technologies. The first optical security element used on Visa credit cards was a relief hologram with the original recorded on the optical table using an analogue technique^3^. This optical element formed a 3D image in the first order of diffraction. At the same time, optical security elements also appeared on banknotes.

The first optical security element on banknotes was computer synthesized^4,5^. The microrelief of the optical element consisted of fragments of binary diffraction gratings. Currently, flat relief security elements protect most banknotes, passports, credit cards, and documents against counterfeiting. Most security elements are computer synthesized^3^.

The microrelief of flat optical elements that form 2D and 3D images is synthesized using various technologies. The microrelief can be formed using both electron-beam and laser technology. Resolution is an important factor for the formation of the microrelief structure at optical wavelengths. Modern facilities for synthesizing optical elements using laser microrelief recording have a resolution of 0.5 microns at best^6,7^, which is insufficient for the formation of asymmetric microrelief of optical elements that produce 3D full-parallax images in the vicinity of zero order. In this study we form asymmetric microrelief of nano-optical elements using electron-beam technology with a resolution of 0.1 micron^8,9^. The latter makes it possible to synthesize nano-optical elements that cannot be forged using widespread methods of microrelief formation based on optical recording techniques.

Electron-beam lithography methods have made it possible to synthesize protective nano-optical elements with multilevel microrelief, which form different easily visually controlled 2D images^10,11^, e.g., a nano-optical element that forms different 2D images when turned by 180°^12^.

Electron-beam technology has already been used to synthesize nano-optical elements that form 3D images^13,14^. In the above studies, a 3D image was formed in the first order of diffraction with the microrelief of the optical element shaped using symmetrical structures. The resulting 3D images can be observed only within a limited range of viewing angles near the first order of diffraction when the element is tilted left/right and up/down; however, the 3D image disappears when the element is rotated.

We discuss the possibilities of synthesizing nano-optical elements to form 3D images in the zero order of diffraction. This is the first time that methods of synthesizing nano-optical elements to form 3D images in the zero order of diffraction have been developed. For such elements, the 3D effect can be observed near the zero order of diffraction both by tilting the element and rotating it by 360°. We develop for the first time methods for computing the phase functions of such nano-optical elements. We use electron-beam lithography to synthesize a microrelief with a 10-nm accuracy in height. The elements that we developed are reliably protected against counterfeiting and can be replicated. Nano-optical elements can be used to protect banknotes, passports, credit cards, etc. against counterfeiting.

## Formulation of the problem of the synthesis of nano-optical elements for the formation of 3D images in the zero order of diffraction and a method for computing the angular patterns in elementary areas

**Figure 1:**
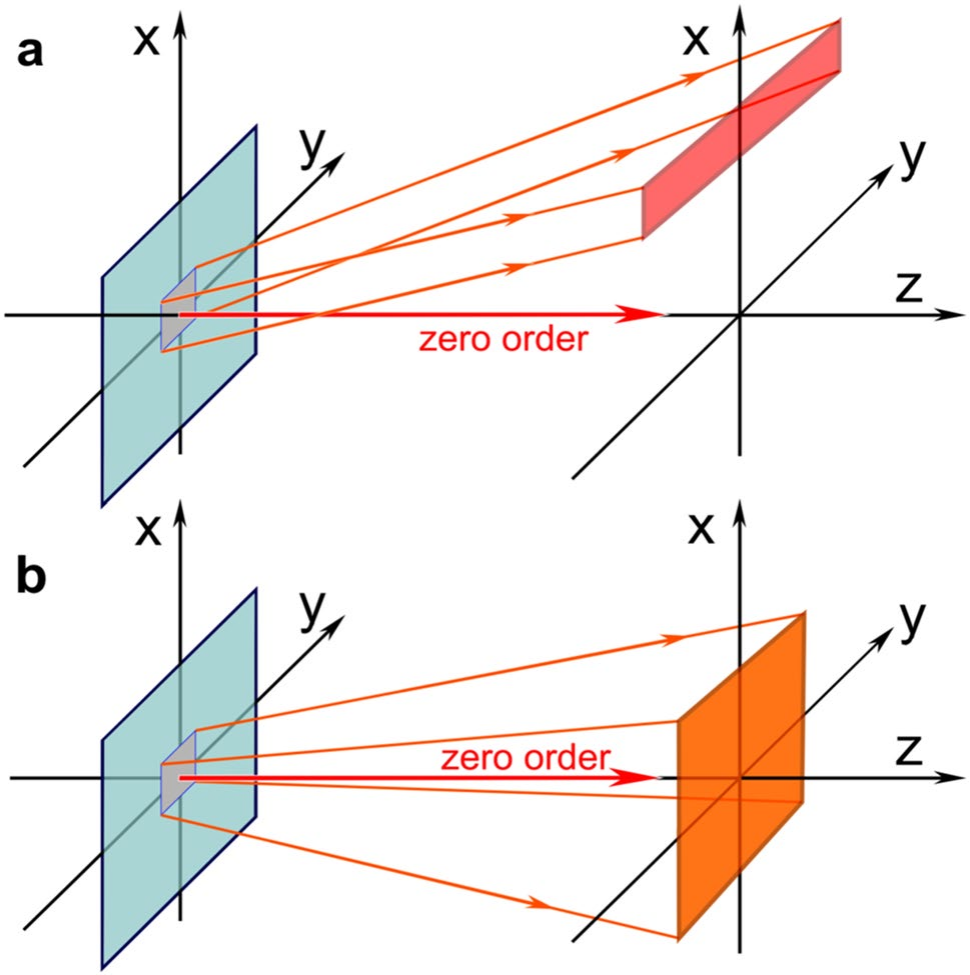
Scheme for observing 3D image: (**a**) formed by rainbow hologram and (**b**) in the vicinity of zero order.

The standard scheme for observing a 3D image formed by a rainbow hologram^2,3^ is shown in Fig. 1a, where the area of observation (the area in which the observer's eyes can be located) is indicated by the red rectangle. The 3D image is formed in the first order of diffraction, and the observation area is a limited narrow band. When the optical element is slightly tilted or rotated, observer’s eyes leave the area of observation and the 3D image disappears for an observer. Figure 1b shows the observation scheme of a 3D image that is formed in the vicinity of the zero order of diffraction, where the observation area is marked in orange. The observation area is a large square centered on the zero order. As long as observer's eyes are within this region, an observer sees a 3D image, and the 3D image is observed over a wide range of tilt angles and even when the optical element is rotated through a full range of 360°.

**Figure 2:**
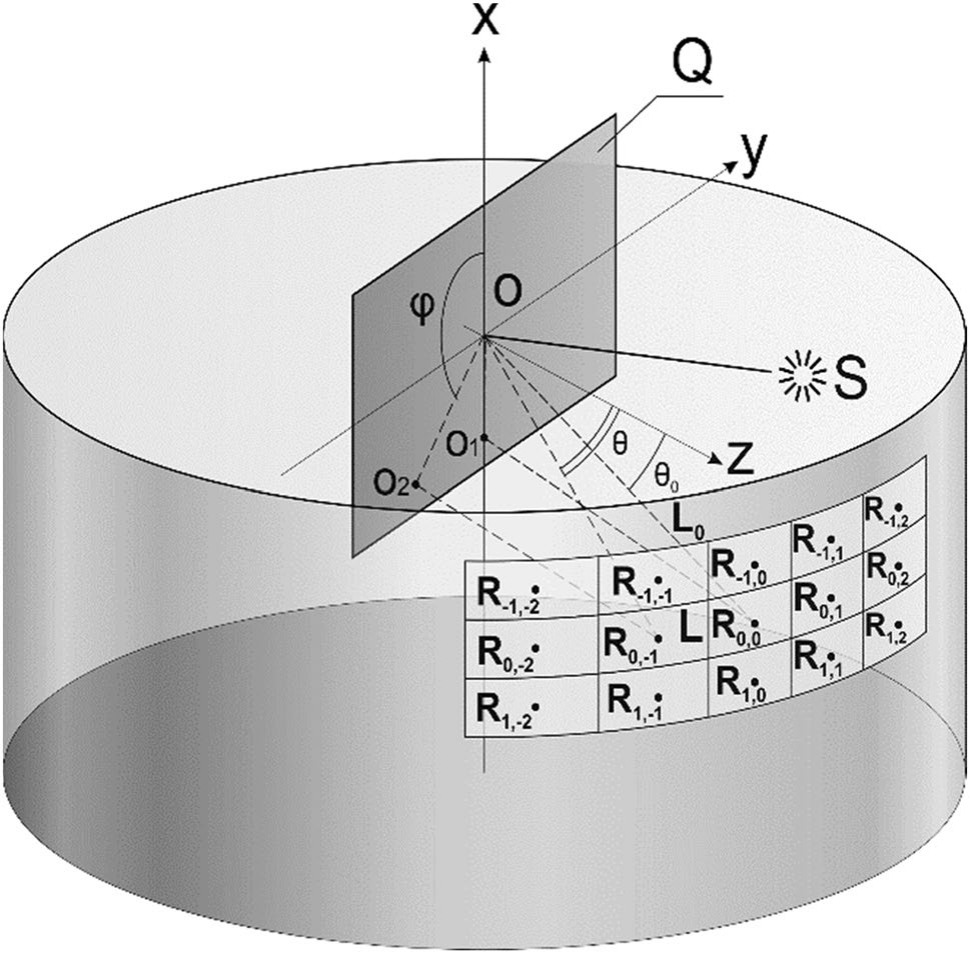
Schematic diagram of the formation of 3D images by a flat optical phase element.

Figure 2 schematically shows the formation of 3D images by a planar reflecting optical phase element at diffraction angles within plus or minus 30° of the zero order.

The optical element is located in the *z* = 0 plane. Figure 2 shows a fragment of observation points (five points along the horizontal direction and three points along the vertical direction). The centres of observation points are marked with the letter “R”. The number of frames is several hundred for real optical elements that form a zero-order 3D image. The radiation source S is located in the Oxz plane of the Cartesian coordinate system. The source is located at an angle θ_0_ to the Oz axis. The direction to the zero order is denoted as L_0_. At different angles φ, θ, the observer sees different 2D frames K_n_, n = 1… N of 3D images. Here, φ and θ are angles in the spherical coordinate system. The angle θ is counted from the Oz axis, and φ is the azimuthal angle. The ray L in Fig. 2 points towards one of the observation points and has angular coordinates φ, θ. Let us assume that the angles (φ_n_, θ_n_) set the directions towards the observation point of frame K_n_, n = 1… N.

**Figure 3:**
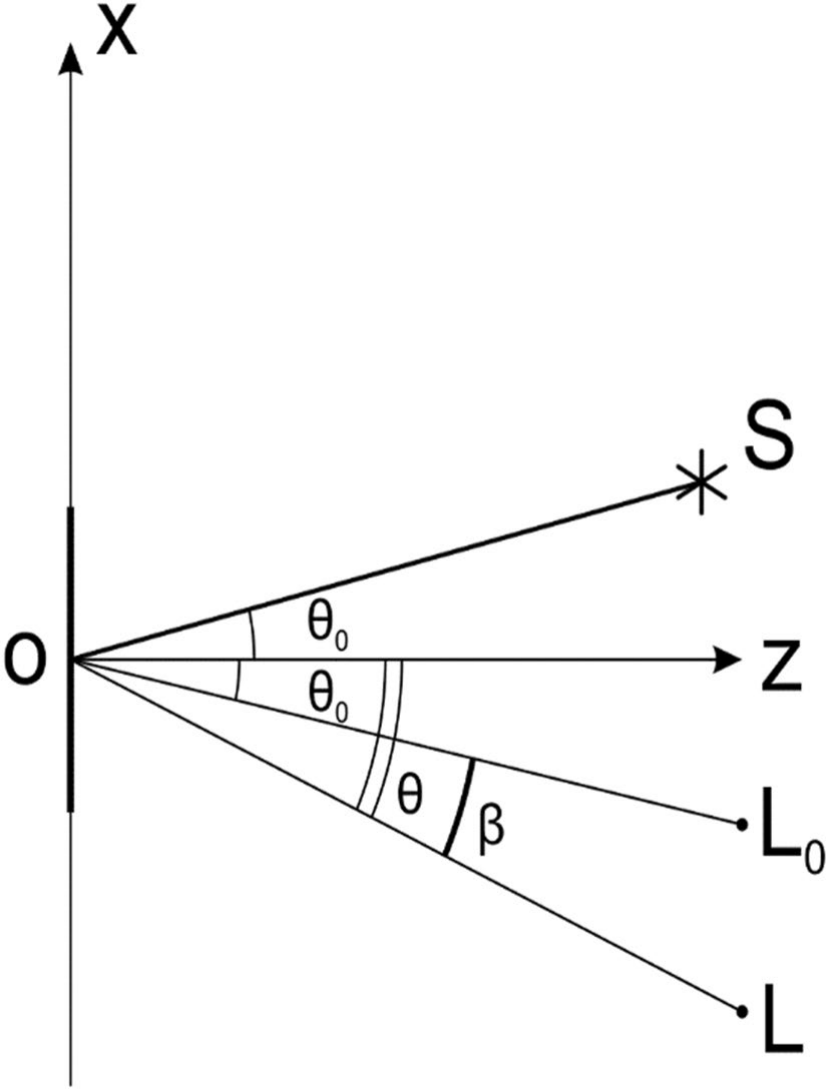
Scheme of observation at small diffraction angles.

Figure 3 shows the observation scheme in the Oxz plane at small diffraction angles. The diffraction angle is defined as the angle between the zero order of diffraction and the direction towards the observation point. Let us denote the diffraction angle as β. For small diffraction angles, the angle is defined by the formula β = θ − θ_0_. A 3D image is observed at diffraction angles within 30° of the zero order of diffraction. The angle θ_0_ between the radiation source S and the normal to the plane of the optical element coinciding with the Oz axis in the diagram determines the zero-order diffraction by beam L_0_.

In this study, for the first time, we develop methods for synthesizing 3D images in the zero order of diffraction. Synthesizing a nano-optical element to form zero-order 3D images is quite a challenging task.

**Figure 4:**
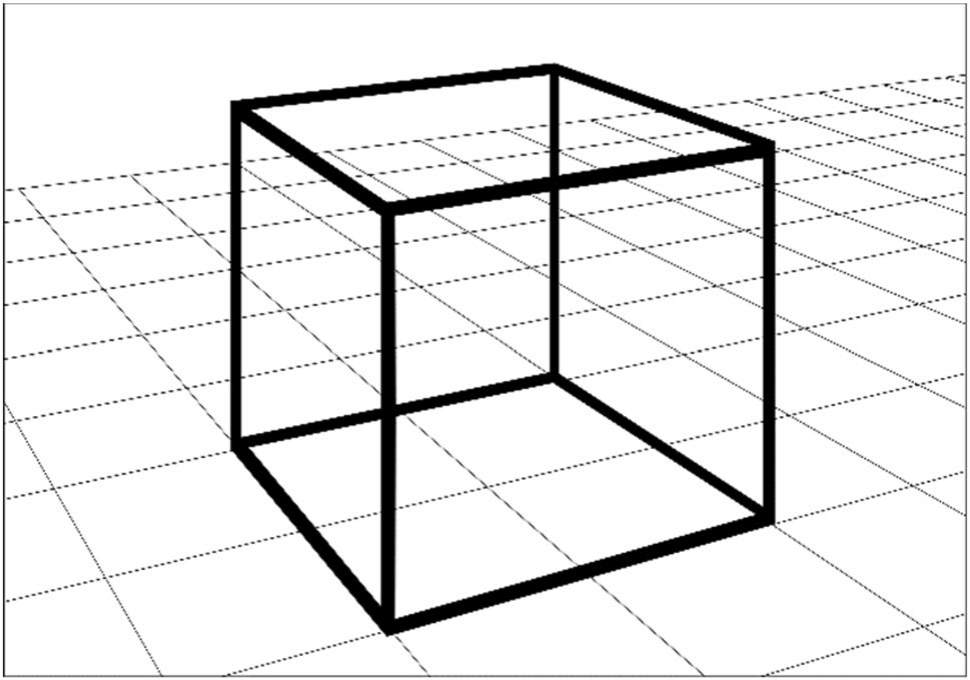
3D model of the object.

The method that we propose in this paper allows the use of different 3D models to form 3D greyscale images. To easily demonstrate the method for calculating the phase function of the diffractive optical element (DOE) a simple 3D object is chosen. Figure 4 shows a computer 3D model of the object, which consists of the edges (coloured black) of a regular quadrilateral cube. This is referred to as a 3D wire-frame model.

**Figure 5:**
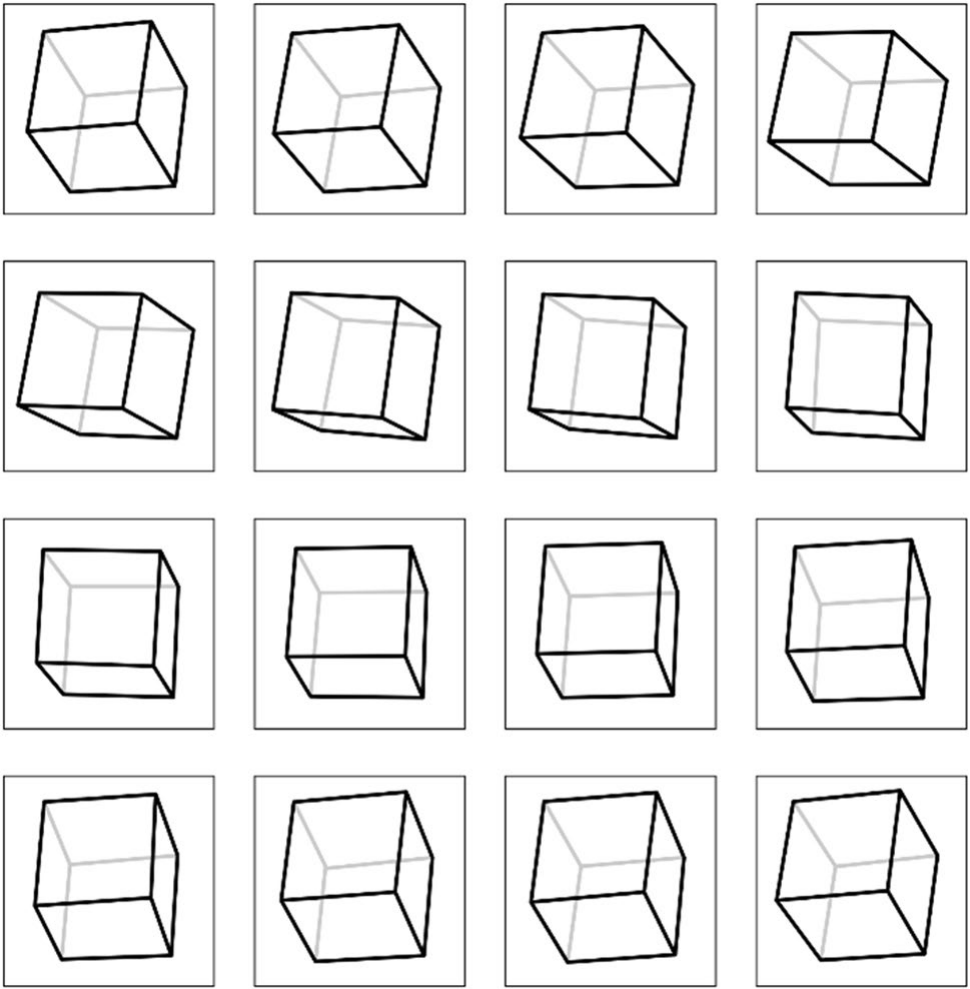
Fragment of the 2D-frames of the 3D-object.

**Figure 6:**
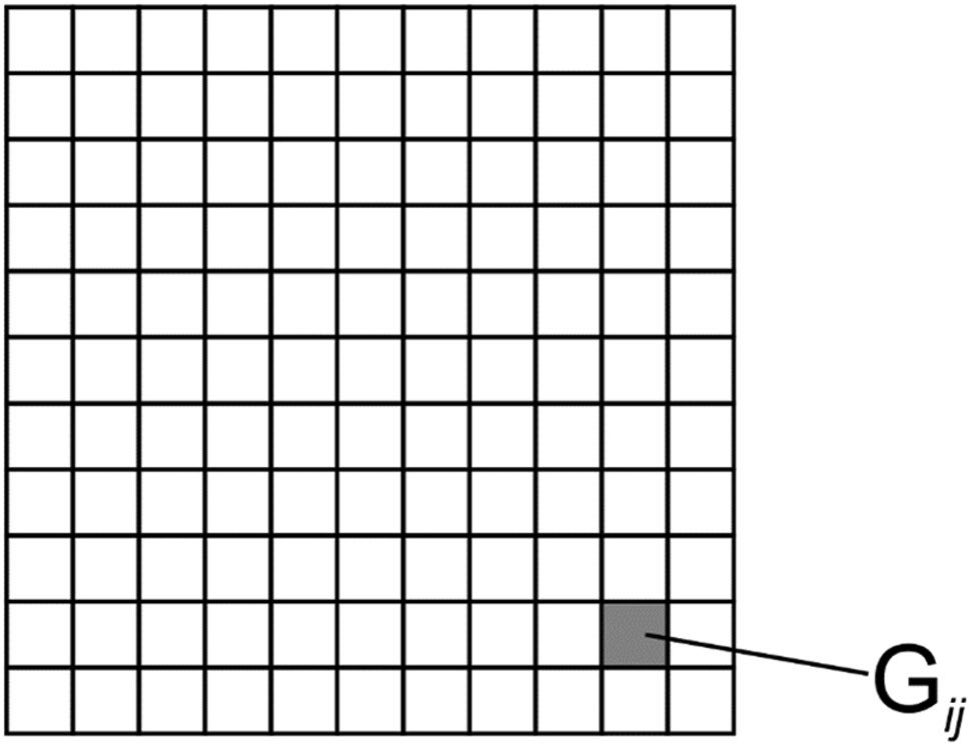
Schematic diagram of the partitioning of an optical element into elementary regions.

Figure 5 shows a fragment of the 2D frames of a 3D object, and Fig. 6 presents a scheme of partitioning the optical element into elementary regions (G_ij_). The size of the elementary region does not exceed 100 microns, which is beyond the resolution of the human eye.

**Figure 7:**
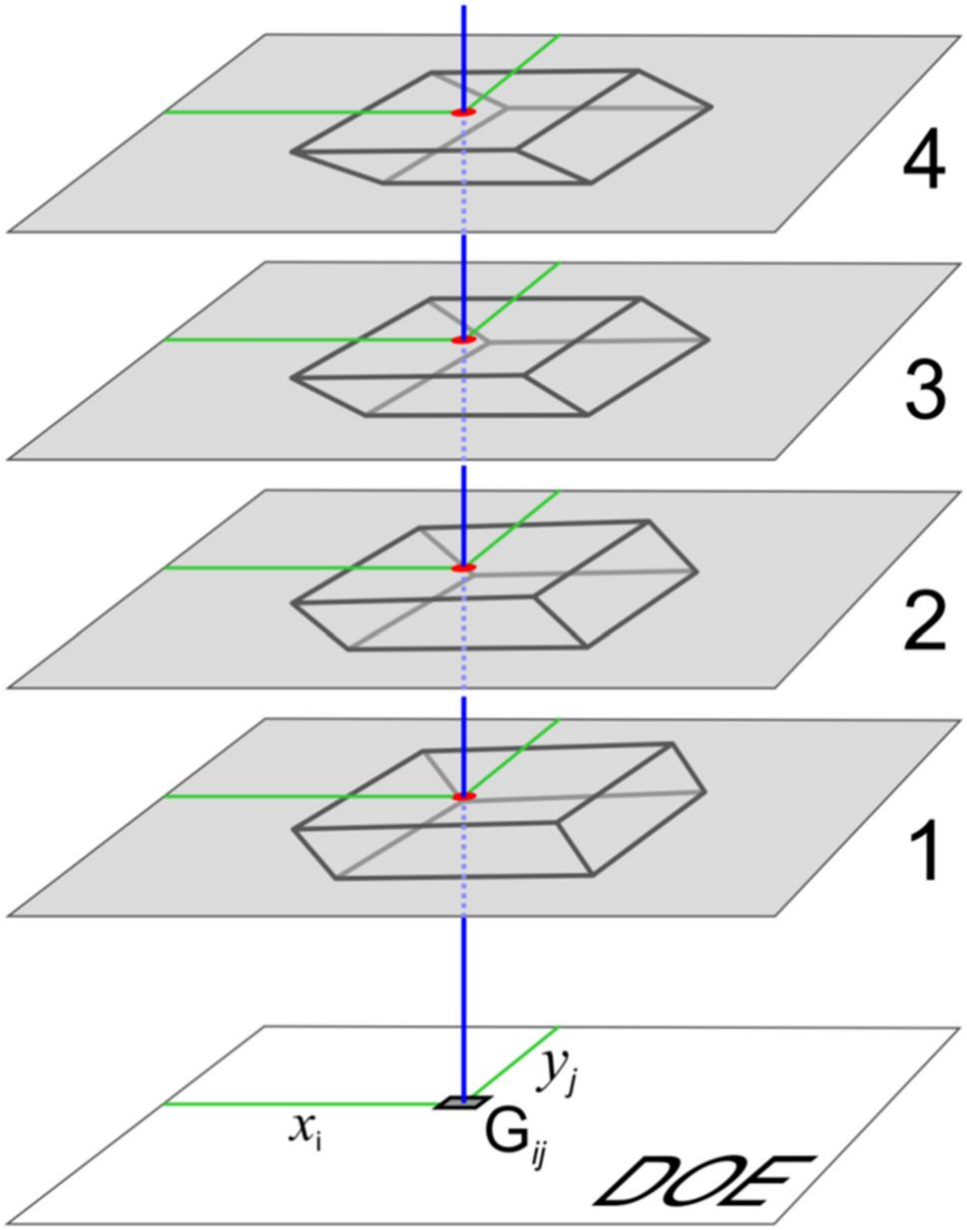
Schematic diagram of the formation of the angular pattern of the area G_ij_.

Figure 7 schematically shows the formation of the angular pattern in the elementary area G_ij_ i = 1… L, j = 1… M. The formation involves all rays from the centre of the elementary area to all observation points (R). The ray L_n_ directed towards the centre of the observation point K_n_ is defined by the angles φ_n_, θ_n_. The number of rays coincides with the number of 2D frames of the 3D image and is equal to several hundred. The intensity of beam L_n_ in the direction (φ_n_,θ_n_) for each n, n = 1… N, is determined as follows. The brightness of point (*x*_*i*_,*y*_*j*_) in frame K_n_ determines the intensity of beam L_n_. As is evident from Fig. 7, in the frames, the intersection point of the 1st and 2nd planes is located in the image and that of the 3rd and 4th planes is located in the background. The size of the elementary area is no greater than 100 µm, and the eye sees this area as a point.

**Figure 8:**
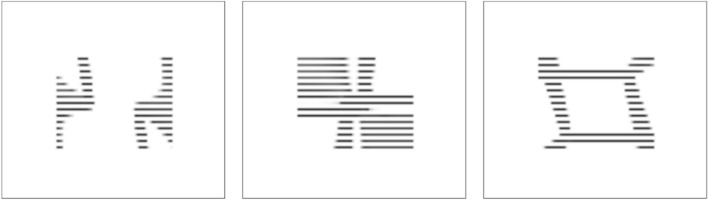
Angular patterns of three different areas G_ij_.

The angular pattern of the radiation scattered from each elementary region G_ij_ is formed at all observation angles (*φ*_n_, *θ*_n_) of the 3D image. Here, n = 1… N. The angular pattern of the region G_ij_ is a set of N rays, and each ray L_n_ has a given intensity. Figure 8 shows the angular patterns computed for three elementary G_ij_ regions.

In the next step, we use the given angular pattern to compute the phase function of the optical element for each elementary region G_ij_.

## Method for computing the phase functions in elementary regions of a nano-optical element

We use the scalar Fresnel wave model to compute the phase functions in the elementary G_ij_ regions. In this model, the scalar wave field $u\left( {x,y,f} \right)$ in the *z* = *f* plane is related to the scalar wave field $u\left( {\xi ,\eta ,0 - 0} \right)$ by the following formula:

1 $$\gamma {\iint_{{G_{ij} }} {u\left( {\xi ,\eta ,{0} - {0}} \right){\text{exp}}\{ ik\frac{{\left( {x - \xi } \right)^{{2}} + \left( {y - \eta } \right)^{{2}} }}{{{2}f}}\} d\xi d\eta } = u\left( {x,y,f} \right){.}}$$

**Figure 9:**
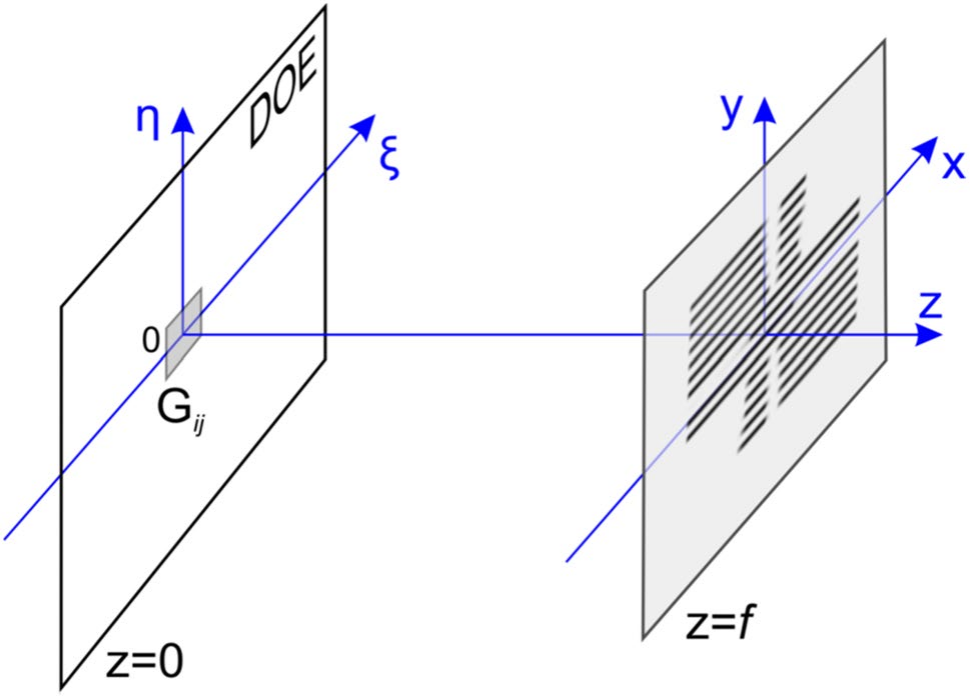
Optical scheme of the formation of the image.

Here *k* = 2π*/λ* and γ = exp(i*kf*)/i*λf* is a given constant where *λ* is a wavelength. Figure 9 shows a scheme of the formation of the 2D image formed by the angular pattern of the elementary region G_ij_ of the flat optical element. The plane wave falls onto the reflecting flat phase optical element whose microrelief forms an image in the *z* = *f* plane.

The peculiarity of the inverse problem of forming a 2D image is that the right-hand side of Eq. (1) does not contain the wave function $u\left( {x,y,f} \right)$ but only its absolute value $F\left( {x,y} \right) = \left| {u\left( {x,y,f} \right)} \right|$.

Let us represent the wave function on the plane z = 0 in the form $u\left( {\xi ,\eta ,0} \right) = \overline{u}\left( {\xi ,\eta } \right)\exp \left( {ik\varphi (\xi ,\eta )} \right)$. Here $\overline{u}\left( {\xi ,\eta } \right)$ is the amplitude and *φ(ξ,η)* is the phase function of the planar optical element in the elementary region G_ij_, We thus have the following operator equation:

2 $${\text{A}}\varphi \left( {\xi ,\eta } \right) \, = F\left( {x,y} \right).$$

In the Fredholm operator equation of the first kind (2) *F*(*x*,*y*) is a given function. The operator A is defined by the following relation:

3 $${\text{A}}\varphi = \left| {\left. {\gamma \iint_{{G_{ij} }} {\overline{u}\left( {\xi ,\eta } \right){\text{exp}}\left( {ik\varphi \left( {\xi ,\eta } \right)} \right){\text{exp}}\left\{ {ik\frac{{\left( {x - \xi } \right)^{2} + \left( {y - \eta } \right)^{2} }}{2f}} \right\}d\xi d\eta }} \right|.} \right.$$

Equation (3) is a nonlinear operator equation with respect to the desired function φ*(ξ,η)* and describes an ill-posed problem^15^. Efficient numerical algorithms have been developed to solve ill-posed linear and nonlinear problems^16,17^. However, one of the most efficient techniques for the approximate solution of Eq. (2) is the method proposed by Lesem et al.^18^. This method later came to be called the Gerchberg–Saxton algorithm^19^. Many studies have been dedicated to investigating this algorithm^20–22^, which, e.g., was shown to be relaxational^23^ and a version of the gradient method for minimizing the functional $R(\varphi ) = \left( {{\text{A}}\varphi - F} \right)^{2}$. There are other variants of gradient minimization of the functional $R(\varphi )$^24^. All these methods have the same property. The value of the functional decreases monotonically quite rapidly during the first 10–20 iterations, and then the decrease rate falls off rapidly.

We follow Lesem et al.^18^ to suggest an algorithm for the approximate solution of nonlinear Eq. (2). Let us introduce the following notation:

4 $${\Phi }\left\{ \nu \right\}\left( {x,y} \right) = \gamma \iint\limits_{{G_{ij} }} {\nu \left( {\xi ,\eta } \right)\cdot\exp \left( {ik\frac{{\left( {x - \xi } \right)^{2} + \left( {y - \eta } \right)^{2} }}{2f}} \right)d\xi d\eta }.$$

Here $\Phi \left\{ \nu \right\}\left( {x,y} \right)$ is the Fresnel transform of function *v*. We construct the iterative process of building the phase function that is an approximate solution of inverse problem (3) as follows. Four steps have to be taken to perform one iteration in the iterative algorithm for solving problem (3). Let *v*^(k)^(*x*,*y*) be given at the *k*-th iteration. We write function *v*^(k)^(*x*,*y*) in the form *v*^*(k)*^*(x,y)* = *A*_0_exp*(ikφ*_*0*_^*(k)*^*(x,y))* and function *w*^(k)^(*x*,*y*) in the form *w*^*(k)*^*(x,y)* = *A*_1_exp*(ikφ*_*1*_^*(k)*^*(x,y))*. Both *A*_0_ and *A*_1_ are real functions. Let *A*_*0*_(*x,y*) be the given intensity distribution of incident light in the *z* = 0 plane. As we are considering phase only diffractive element, then the amplitude *A*_*0*_(*x,y*) in our case will be equal to one in the elementary region G_ij_. Let *A*_*1*_(*x,y*) = *F*(*x,y*) be the given intensity distribution in the focal plane *z* = *f*. The algorithm for solving the inverse problem consists of the following four steps performed in sequence:

5 $$\begin{array}{*{20}l} {1)} \hfill & {\varphi_{1}^{\left( k \right)} \left( {x,y} \right) = \arg \left( {{\Phi }\{ v^{\left( k \right)} \} \left( {x,y} \right)} \right)} \hfill \\ {2)} \hfill & {w^{\left( k \right)} \left( {x,y} \right) = A_{1} \left( {x,y} \right)\exp \left( {ik\varphi_{1}^{\left( k \right)} \left( {x,y} \right)} \right)} \hfill \\ {3)} \hfill & {\varphi_{0}^{{\left( {k + 1} \right)}} \left( {x,y} \right) = \arg \left( {{\Phi }_{{}}^{ - 1} \{ w^{\left( k \right)} \} \left( {x,y} \right)} \right)} \hfill \\ {4)} \hfill & {v^{{\left( {k + 1} \right)}} \left( {x,y} \right) = A_{0} \left( {x,y} \right)\exp \left( {ik\varphi_{0}^{{\left( {k + 1} \right)}} \left( {x,y} \right)} \right)} \hfill \\ \end{array}$$

The function $\varphi_{0}^{{\left( {k + 1} \right)}}$ is an approximate solution of Eq. (2). A phase distribution equal to a constant can be used as an initial approximation. The phase function *φ(ξ, η)* computed by the iterative process (5) uniquely determines the microrelief in the region G_ij_. For example, for a normal wave incident on an optical element, the depth of the microrelief in the region G_ij_ is equal to 0.5 *φ(ξ, η)* for any point *(ξ, η)* in this region.

**Figure 10:**
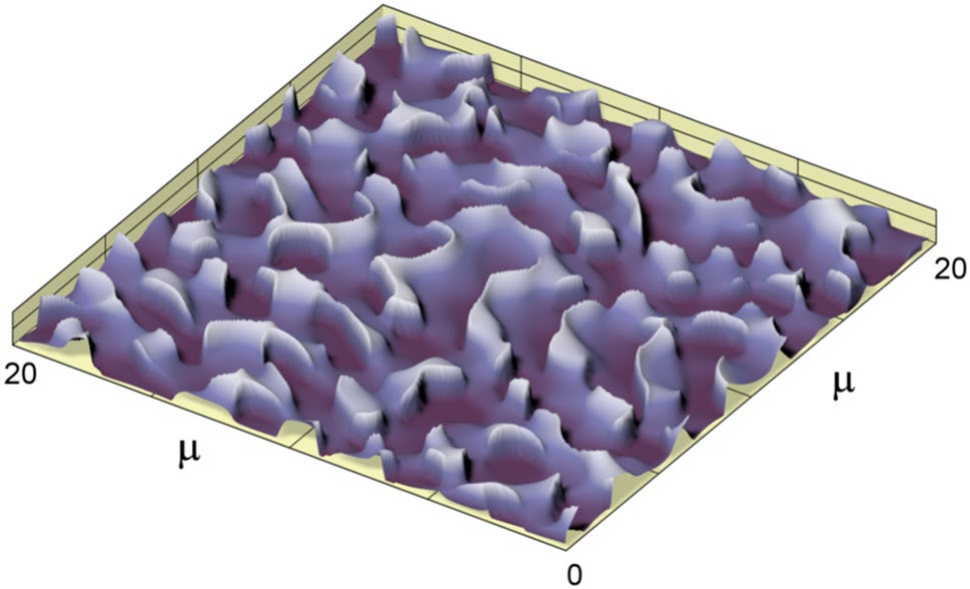
Fragment of the microrelief of the multilevel kinoform in one of the elementary G_ij_ regions.

Figure 10 shows a fragment of the microrelief of the multilevel kinoform in one of the elementary G_ij_ regions. The image size is 20 × 20 µm^2^. The depth of the microrelief does not exceed 0.5λ and is equal to approximately 300 nm.

Thus, the solution of the inverse problem for each elementary region G_ij_, i = 1… L, j = 1… M yields the microrelief on the entire area of the nano-optical element. The above algorithm for computing the phase function can be applied to the 3D model of any 3D object.

## Examples of the synthesis of multilevel nano-optical elements for zero-order 3D imaging

### Example 1

To demonstrate the efficiency of the proposed technologies, we made a 16 × 16 mm^2^ nano-optical element to form a zero-order 3D image. The 3D image consists of the edges of a regular cube. We used multilevel kinoforms to produce the 3D image. A 16 × 16 mm^2^ flat optical element was partitioned into 160,000 50 × 50 μm^2^ elementary G_ij_ regions, i = 1… L, j = 1… M, as shown in Fig. 6. The number of frames N was 825 (55 frames horizontally, 15 frames vertically). We compute the microrelief of the flat optical element at the given wavelength λ = 547 nm for each elementary region G_ij_. To compute the phase function in the area G_ij_, we use a 500 × 500 grid to solve the inverse problem (2) of computing the phase functions in the elementary regions, and it takes more than 10 min to compute the phase function for the entire optical element on a PC.

We used an electron-beam lithography system with a variable beam shape and a minimum beam size of 0.1 μm to produce the microrelief of the nano-optical element and used a positive electron resist to record microstructures (see Supplementary Information for details). The maximum microrelief depth was 0.3 μm, and the depth accuracy of microrelief formation was 10 nm. The original nickel master matrix of the diffractive optical element was made using a standard electrotyping procedure.

**Figure 11:**
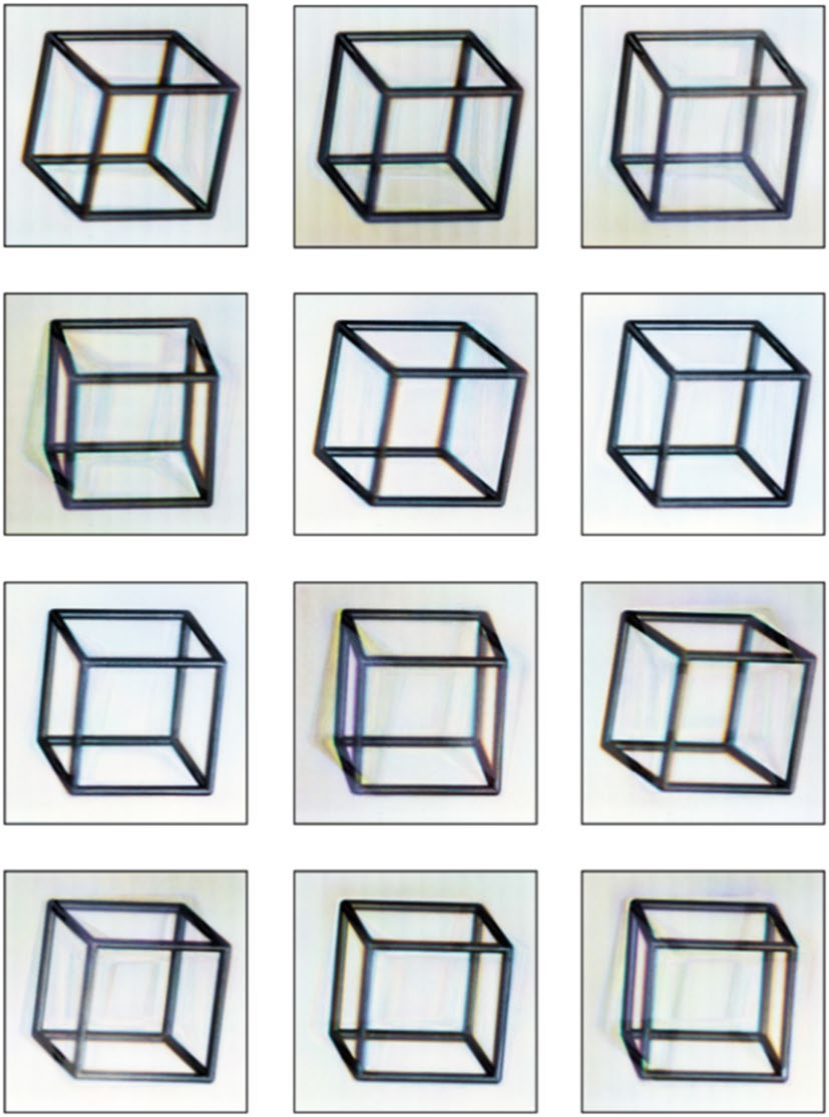
3D images of the cube at different angles (see Supplementary Video S1 and Supplementary Video S2).

Figure 11 shows photographs of the nano-optical element taken from different viewing angles at diffraction angles of plus or minus 30° relative to the zero order of diffraction. A cell phone flash was used as the white light source.

We computed the microrelief at a wavelength of λ = 547 nm, which corresponds to green light, but the quality of the images formed remains good even if the element is illuminated with white light. As is evident from Fig. 11, the nanooptical element forms an image of a cube, but inaccuracies in the formation of the microrelief of the optical element result in very low-intensity ghosts that show up against white background. However, such ghosts become invisible if a 3D model forming lower-contrast frames is used. We demonstrate this in Example 2.

### Example 2

**Figure 12:**
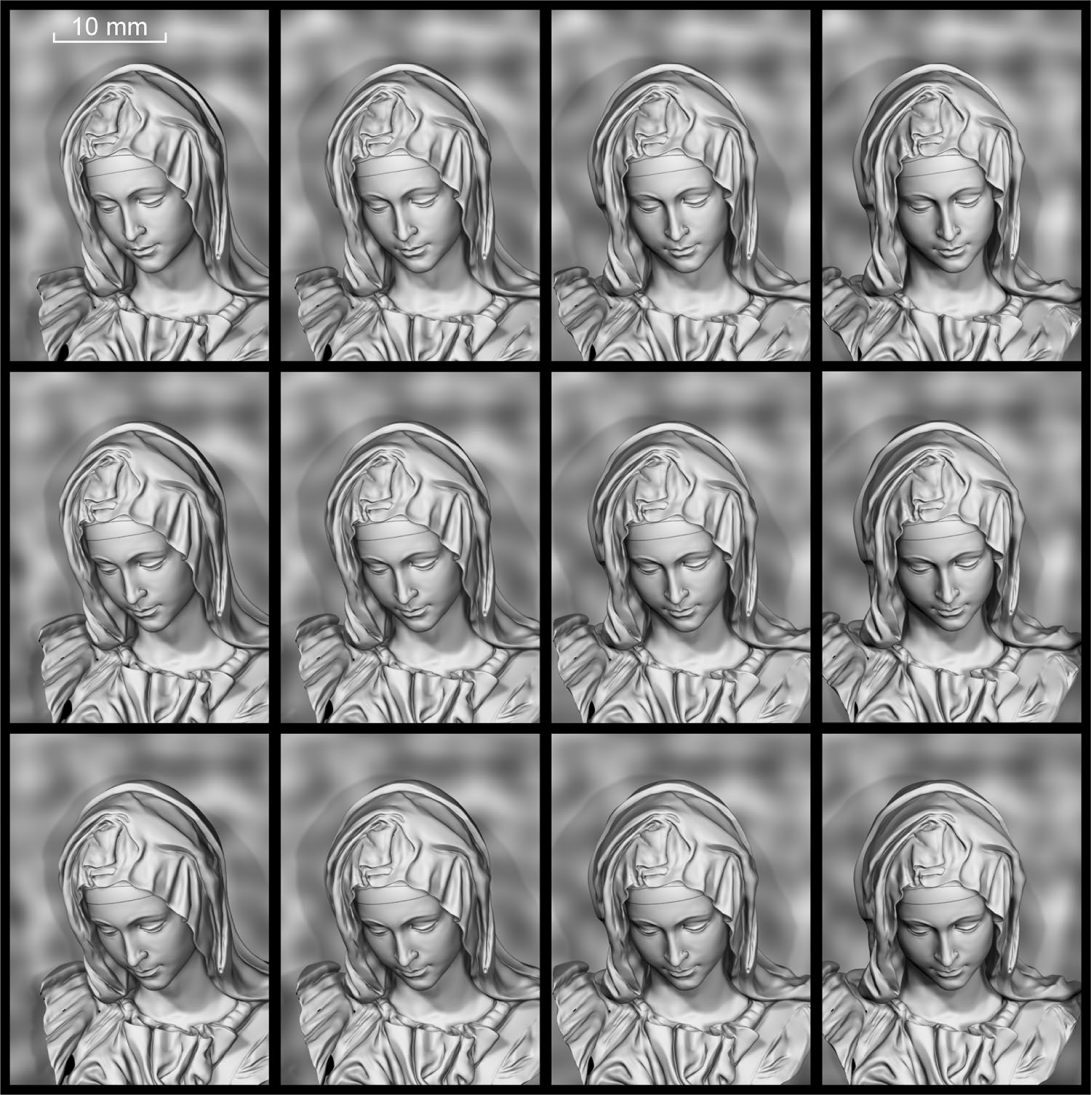
Fragment of the 2D-frames of the 3D-object.

In example 2, the problem of the synthesis of a nano-optical element is considered, which forms a greyscale 3D image of a complex shape when illuminated with a white light source. Figure 12 shows 12 2D frames obtained by rendering a computer 3D model. The observer should see different 2D frames of a given 3D image from different viewing angles. For the 3D image in example 2, 1440 2D frames were used.

**Figure 13:**
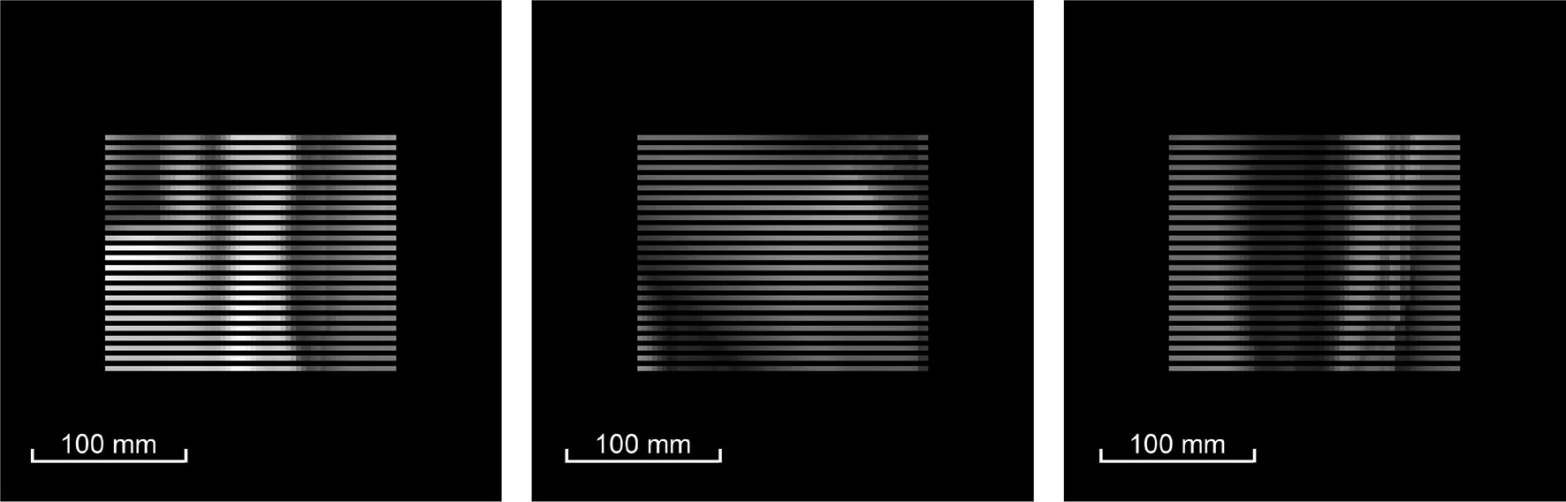
Intensity distribution in the focal plane (f = 300 mm) for three different elementary regions G_ij_.

The area of the nano-optical element was divided into elementary G_ij_ (i = 1… L, j = 1… M) regions, with a size of 50 × 50 μm^2^, as shown in Fig. 6. The size of the nano-optical element was 25 × 35 mm. Acting according to the algorithm described in paragraph 2 using 2D frames (Fig. 12) for each elementary area G_ij_, i = 1… L, j = 1… M, the beam patterns were calculated in each of the elementary areas. Figure 13 shows beam patterns for three elementary regions.

We compute the microrelief of the flat optical element at the given wavelength λ = 547 nm for each elementary region G_ij_. To calculate the phase functions in the elementary G_ij_ regions from the calculated radiation patterns, a 500 × 500 grid was used. The phase function uniquely determines the microrelief of the nano-optical element.

**Figure 14:**
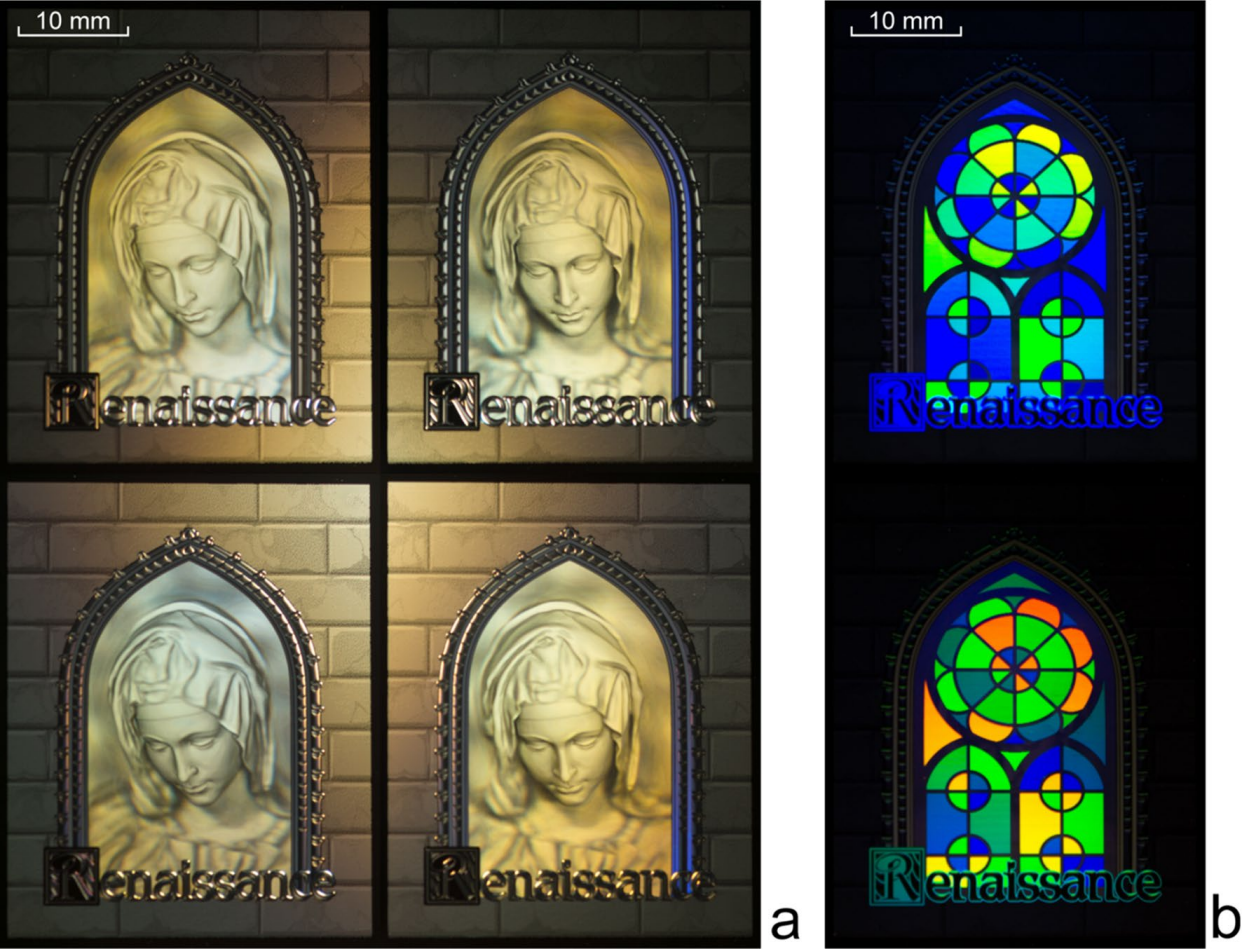
Photos and videos (see Supplementary Video S3 and Supplementary Video S4) of Example 2 at different positions of the light source and a fixed position of the sample and camera: (**a**) a 3D image is observed at diffraction angles within 30° and (**b**) a 2D colour image is visible at diffraction angle greater than 60°.

Figure 14 shows photographs of the nano-optical element taken from different viewing angles. The structure of an optical element forming a 3D image in the zero order of diffraction can be modified to make the kinoform fill the G_ij_ regions partially rather than completely.

The structure of an optical element forming a 3D image in the zero order of diffraction can be modified to make the kinoform fill the G_ij_ regions partially rather than completely. The remaining parts of the elementary G_ij_ regions can be filled with diffraction gratings with periods less than 0.7 μm. These diffraction gratings can form an additional 2D colour image visible to the observer over the entire area of the optical element at diffraction angles greater than 60°.

## Discussion and conclusion

In this paper, we develop methods for synthesizing nano-optical elements to form 3D images at the zero-order diffraction for the first time. The synthesis methods include both the computation of the phase function of the nano-optical element and the formation of its microrelief by means of electron-beam lithography. From a mathematical point of view, the computation of the phase function is a typical inverse problem, which we solve in two steps. In the first step, we use all the image frames that define a 3D object to generate the angular patterns in each elementary region. In the second stage, we compute the phase functions of the nano-optical element in each elementary region. The latter problem reduces to solving a nonlinear integral equation. Despite the large number of elementary regions (~ 300,000), a personal computer is sufficient to compute the phase function.

We used electron-beam lithography to form the microrelief. The accuracy of microrelief formation is 10 nm in height. We produced a sample nano-optical element that forms a 3D image in the zero order of diffraction. The resulting 3D image can be observed when illuminated by white light, and the observer sees a 3D image with full parallax both when tilting the optical element and when rotating it by 360°. A 3D image can also be formed in the first order of diffraction, as we did, for example, in our earlier study^13^ using a binary microrelief. In this case the diffraction efficiency of the optical element does not exceed 40%. The use of multilevel microrelief makes it possible not only to increase the diffraction efficiency but also to significantly widen the viewing angles of the 3D image.

The nano-optical element can be replicated using standard equipment for the production of relief holograms. The synthesis methods developed are designed to protect banknotes, passports, and plastic cards against counterfeiting. The technology of the synthesis of nano-optical elements is knowledge intensive and not widespread, thereby ensuring bona fide protection of the developed elements against counterfeiting.

Methods for computing the phase functions of nano-optical elements can be used in prospective 3D displays and 3D projectors. Currently, supercomputer technology is widely used to accelerate computations. The phase function in each elementary region is computed independently, allowing the algorithms to be easily parallelized. The use of a graphics processing unit (GPU) cluster can accelerate the computation of the phase function of a nano-optical element by hundreds or thousands of times. Currently, processors with eight hundred thousand cores are developed and available on the market^25^. The use of such technologies can make it quite possible to compute the phase function of the entire optical element in a fraction of a second, thereby opening up opportunities for the synthesis of animated 3D images in prospective 3D design systems and 3D displays^26,27^.

## Methods

The synthesis method developed in the article includes the calculation of the DOE phase function, which has the effect of forming 3D images with full parallax at the zero order of diffraction. The synthesis of the DOE includes the calculation of its phase function and the fabrication of the microrelief. The calculation of the phase function is carried out in two stages. In the first stage, the DOE is divided into elementary regions of 50 microns in size. We created two nanooptical elements (Example 1 and Example 2) for forming 3D images with full parallax at the zero order of diffraction. The total number of elementary regions for Example 1 produced with a size of 16 × 16 mm is 102,400. The total number of elementary regions for Example 2 produced with a size of 25 × 35 mm is 350,000. The radiation pattern of the reflected light is calculated for each elementary region. Each elementary region is involved in the formation of all K_n_ frames. The total number of frames for Example 1 used is 825. The total number of frames for Example 2 used is 1440. For the calculation, the scheme shown in Fig. 7 is used. The radiation pattern of the reflected light is calculated in the geometrical approach in the finite parametric model. In the second stage, for each elementary region, the DOE phase function is calculated according to a given radiation pattern. For the calculation, algorithm (5) is used; to increase the calculation speed, a fast Fourier transform is implemented. The total time of calculation of the entire phase function for Example 1 is 140 s for radiation patterns in all elementary regions plus 80 min for the kinoforms in all elementary regions. The total time of calculation of the entire phase function for Example 2 is 140 s for radiation patterns in all elementary regions plus 270 min for the kinoforms in all elementary regions. All computations are carried out on a PC with AMD Phenom II X6 3.2 GHz CPU and 16 Gb DDR3 memory. The phase function uniquely determines the microrelief of the DOE. The calculations are performed for a fixed wavelength of 547 nm, and the microrelief is formed by using electron beam lithography. A shaped electron beam lithography system is used to form the microrelief, where the minimum beam size is 0.1 × 0.1 microns and the maximum possible beam size is 6.3 × 6.3 microns. The microrelief is formed on a positive PMMA resist with a thickness of 0.5 micron, and the accuracy of the formed microrelief at depth is not greater than 10 nm.

Then, the microrelief is coated with a thin layer of silver using a vacuum evaporation system with a resistive thermal heater. Next, a 0.2 mm-thick nickel master shim is grown in an electroforming bath, and the master shim is used to capture photos and videos for the present article.

## Supplementary Information

Supplementary Video 1.

Supplementary Video 2.

Supplementary Video 3.

Supplementary Video 4.

Supplementary Information 1.

## Data Availability

The authors confirm that the data supporting the findings of this study are available within the article and its supplementary materials.
