# Supplementary material for: Synthesis of nano-optical elements for zero-order diffraction 3D imaging
Source: Sci Rep. 2022 May 23;12:8639. doi: 10.1038/s41598-022-12414-y (PMC9126882; doi:10.1038/s41598-022-12414-y)
Supplement: Supplementary file 5 — Supplementary Information 1. [file 41598_2022_12414_MOESM5_ESM.pdf]

Supplementary Information for

Synthesis of nano-optical elements for zero-order diffraction 3D imaging

Alexander Goncharsky[1], Anton Goncharsky[1], Dmitry Melnik[2] and Svyatoslav Durlevich[1]*
[1] Research Computer Center, M.V. Lomonosov Moscow State University, Leninskiye Gory, 1, building 4, Moscow 119991, Russia
[2] Computer Holography Centre Ltd., str.2, Proezd 4922, Zelenograd, Moscow, 124460, Russia
**sdurlevich@ya.ru*

Supplementary Video 1 shows full parallax of the 3D image formed by the DOE (Example 1).
Parameters: White light source is from above and it moves in two directions, fixed position of the DOE, fixed position of the camera.

Supplementary Video 2 shows the 3D image formed by the DOE, when the DOE (Example 1) is rotated through 360 degrees.
Parameters: Fixed position of the camera, white light source has fixed position from above, the nickel shim with the DOE rotates through 360 degrees.

Supplementary Video 3 shows full parallax of the 3D image formed by the DOE (Example 2).
Parameters: White light source is from above and it moves in two directions, fixed position of the DOE, fixed position of the camera.

Supplementary Video 4 shows the 3D image formed by the DOE, when the DOE (Example 2) is rotated through 360 degrees.
Parameters: Fixed position of the camera, white light source has fixed position from above, the nickel shim with the DOE rotates through 360 degrees.

Supplementary Video 4 shows the 3D image formed by the DOE, when the DOE (Example 2) is rotated through 360 degrees.
Parameters: Fixed position of the camera, white light source has fixed position from above, the nickel shim with the DOE rotates through 360 degrees.

Supplementary Material. E-beam lithography for manufacturing of nano-optical elements in Example 1 and 2.

Electron beam lithography is a technology for synthesizing various microstructures with extremely high resolution. The resolution of the record is determined by the minimum size of the electron beam. The electron beam can be focused onto a spot with a size of a few nanometers and this fact determines the highest resolution of e-beam technology. For comparison, even theoretically the minimum size of the focused spot of laser radiation cannot

be smaller than the wavelength. We can thus conclude that e-beam technology exceeds optical methods by two orders of magnitude in terms of resolution.

The principle of recording using e-beam technology can be briefly described as follows. A thin film made of a special material - electron resist - spread over a thin plate is exposed to the electron beam. A computer-controlled electron beam exposes certain areas in accordance with the given program. After exposure, the plate covered by electron resist is subject to a special treatment - the resist developing process. During this process the areas of the resist exposed to the electron beam are treated differently from the unexposed areas. For example, unexposed areas remain on the plate, whereas the areas exposed by the electron beam are removed. As a result, depressions form in the exposed areas. Thus, the treatment of a resist-covered plate creates a high-resolution microrelief on it.

The principal technological process - exposure - is performed by special instruments - electron-beam lithography systems. The latter are extremely complex systems of devices that have absorbed the latest advances of science and technology. Figure 1(a) and (b) shows a block diagram and a photo of the electron-beam system, respectively.

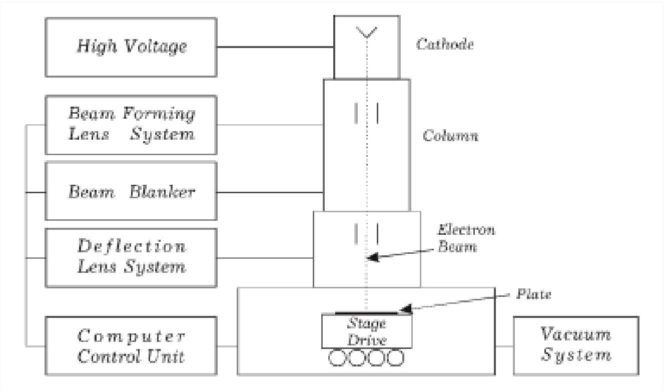

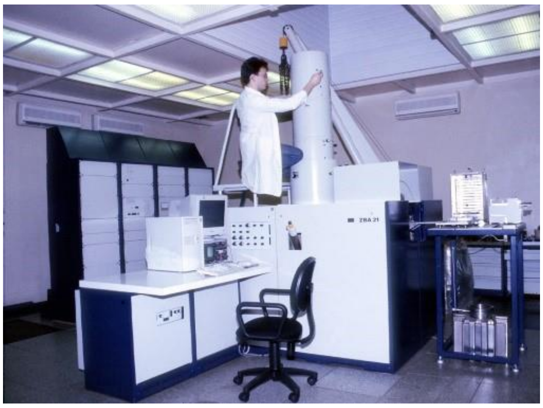

a) b)

Fig 1. Block diagram (a) and photo (b) of the e-beam lithography system

The e-beam system consists of an electron column, beam forming and beam-deflection system, a control system with software, and various auxiliary units (vacuum unit, plate loading/unloading system, etc.). Some models of electron beam systems also allow shaping of the electron beam. The focused electron beam hits the plate covered by a thin film electron-sensitive material. The deflection limit of the electron-beam deflection usually does not exceed 3 mm. Correspondingly, after completing the exposure of a 3 x 3 mm^2 area the stage is shifted to another position and another area is exposed. For microrelief forming in Example 1 и Example 2 authors used shaped beam lithography system ZBA with the minimal beam size of 0.1 x 0.1 micron. Maximum size of electron beam is 6.3 x 6.3 microns. The accuracy of microrelief formation is 10 nanometres in terms of depth. Accelerated voltage of the e-beam system is 20kV. Max. size of e-beam resist plates is 152 mm x 152 mm.

Consider now a typical process of recording the microrelief of an optical element in electron-beam technology (see Fig. 2).

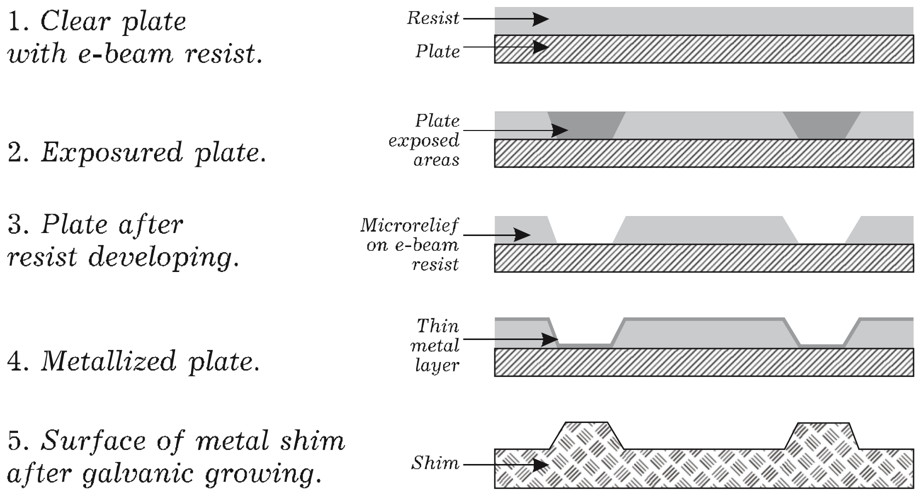

Fig 2. Scheme of e-beam technology of microrelief fabrication

The procedure of forming of a single nickel shim with microrelief consists of a few main stages:
(1) Loading of the resist-covered plate into the system
(2) Exposure process - the electron beam exposes specified areas.
(3) Processing of the resist - exposed areas are removed and the microrelief of the element forms on the resist.
(4) Vacuum application of the reflective coating onto the element. After metallization, we have a reflective conducting surface with microrelief.
(5) Growing of a galvanic replica of the metallized surface.

As a result, we obtain a solid metallic plate with the microrelief of the optical element - a reflecting phase-only optical element. The basic scheme described above allows manufacturing both binary and multilevel elements. For microfabrication PMMA (Polymethyl Methacrylate) positive e-beam resist was used. Thickness of e-beam resist used was 450nm. For metallization of microrelief authors used silvering in vacuum, for galvanic growing of the nickel shim it was used nickel sulfamate electrolyte.
